# Adult granulosa cell tumor associated with endometrial carcinoma: a case report

**DOI:** 10.1186/1752-1947-5-340

**Published:** 2011-08-02

**Authors:** Cornelius O Ukah, Okechukwu C Ikpeze, George U Eleje, Ahizechukwu C Eke

**Affiliations:** 1 Department of Histopathology, Nnamdi Azikiwe University Teaching Hospital, Nnewi, Anambra State, Nigeria; 2 Department of Obstetrics and Gynecology, Nnamdi Azikiwe University Teaching Hospital, Nnewi, Anambra State, Nigeria; 3 Health Policy and Management concentration, Masters in Public Health Program, Harvard School of Public Health, 677 Huntington Avenue, Boston, MA 02115, USA

## Abstract

**Introduction:**

If strict criteria for the diagnosis of carcinoma are used and all patients with granulosa cell tumors are considered, the best estimate of the incidence of associated endometrial carcinomas is under 5%. In patients with granulosa cell tumors, estrogen-dependent endometrial cancers are rarely found, and most of these endometrial cancers are well-differentiated endometrioid adenocarcinomas that carry a good prognosis when detected early.

**Case presentation:**

We report the case of a 65-year-old post-menopausal Nigerian woman of the Igbo tribe with an adult granulosa cell tumor that was initially treated as endometrial carcinoma. She underwent a total abdominal hysterectomy and a bilateral salpingo-oophorectomy after histopathologic confirmation of a well-differentiated granulosa cell tumor of the ovary and a nuclear grade 1 adenocarcinoma of the endometrium (International Federation of Obstetricians and Gynecologists stage 1B). She had a good post-operative recovery and was discharged 10 days after treatment.

**Conclusion:**

The association between adult granulosa cell tumors of the ovary and endometrial carcinomas is rare. A high index of suspicion as well as good imaging and histopathologic analyses are important in making this diagnosis.

## Introduction

Adult granulosa cell tumors account for approximately 1% to 2% of all ovarian tumors and 95% of all granulosa cell tumors [1]. They occur more often in post-menopausal than pre-menopausal women, with a peak incidence between 50 and 55 years of age. They are the most common estrogenic ovarian tumors diagnosed clinically, but the precise proportion of adult granulosa cell tumors that secrete hormones is difficult to establish because an endometrial tissue specimen used to evaluate the effects of estrogenic stimulation is often unavailable. The typical endometrial reaction associated with functional tumors in this category is simple hyperplasia that usually exhibits some degree of pre-cancerous atypicality. Reports of the incidence of an associated endometrial carcinoma, which almost always is well differentiated, have ranged from slightly less than 5% to slightly more than 25% of cases [1,2]. The wide variation in these figures is attributable at least in part to differing views of the dividing line between complex atypical hyperplasia and grade 1 adenocarcinoma. If strict criteria for the diagnosis of carcinoma are used and all patients with granulosa cell tumors, not only those who have undergone endometrial curettage or hysterectomy, are considered, the best estimate of the incidence of an associated endometrial carcinoma is under 5% [1].

## Case presentation

We present the case of a 65-year-old, para 11, Nigerian woman of the Igbo tribe with 10 living children. She was six years post-menopausal and had borne her last child 26 years before presentation to our hospital. She presented with a three-week history of post-menopausal bleeding. The vaginal bleeding was sudden in onset and profuse, with occasional passage of blood clots. She had no history of dizziness or fainting. She had associated intermittent lower abdominal pain that was dull, 4 of 10 in intensity, with no relieving or aggravating factors. She had no history of post-coital or contact bleeding, weight loss, anorexia, urinary symptoms, abdominal mass, vaginal discharge, or dyspareunia. She had no history of the use of hormonal contraceptives or ovulation-inducing drugs. She had no family history of a similar illness. She had attained menarche at the age of 15 years, and her coitarche occurred at age 16 years. She had not had any Papanicolaou smears in the past. She was not known to have diabetes or hypertension, and she neither smoked cigarettes nor drank alcohol.

An endometrial tissue biopsy was done in a private hospital. The histology revealed a well-differentiated endometrial adenocarcinoma. She was subsequently referred to our teaching hospital for further management. Upon presentation to our teaching hospital, her physical examination revealed an elderly woman in no distress who was afebrile (body temperature 37.1°C), not pale, and anicteric. She was not dehydrated. No peripheral lymphadenopathies and no pedal edema were present. Her pulse rate was 90 beats/minute, regular, and with a full volume. Her blood pressure was 170/90 mmHg. S1 and S2 heart sounds were heard, and there were no murmurs, rubs, or gallops. Her jugular venous pressure was not increased. Her respiratory rate was 22 cycles/minute, and her chest was clinically clear with vesicular breath sounds. The findings of the abdominal examination were normal.

The vaginal examination revealed an atrophic, blood-stained vulvovagina. A speculum examination showed an apparently healthy-looking cervix. The cervical os was found to be closed on digital examination. A bimanual examination revealed a bulky uterus that was about 16 weeks' gestational size. It was freely mobile and anteverted. There was no adnexal mass or tenderness, and there was no cervical excitation tenderness.

A clinical diagnosis of post-menopausal bleeding secondary to endometrial carcinoma and essential hypertension was made. She was admitted to the gynecology ward. Her packed red blood cell volume was 33%, and the total white blood cell count was 3.4 × 10^9^/L. The human immunodeficiency virus screening results were negative. The urine analysis, renal function test, liver function test, electrocardiography, and the fasting lipid profile results were all normal. A chest radiograph revealed mild cardiomegaly. Abdominopelvic ultrasonography showed an enlarged uterus with an endometrial thickness of 15 mm. There were no obvious hepatic lesions, and there was no hydronephrosis.

In view of the patient's co-existing essential hypertension, the cardiology team was invited to co-manage the patient. She was prescribed tablet amlodipine (5 mg/day), hydrochlorothiazide (25 mg/day), and tablet aspirin (75 mg/day, including hematinics). She was also counseled regarding total abdominal hysterectomy and bilateral salpingo-oophorectomy that would be performed after her blood pressure had stabilized.

Three weeks after initiation of her anti-hypertension therapy, she underwent a total abdominal hysterectomy and a bilateral salpingo-oophorectomy while under general anesthesia with endotracheal intubation. The intra-operative findings included a bulky uterus that measured 8 cm × 5 cm with a blood-filled cavity, an enlarged, malignant-looking endometrium, and a massive hemoperitoneum from a ruptured soft but malignant-looking huge friable left ovarian mass that measured 5 cm × 5 cm in diameter. Her fallopian tubes and right ovary appeared to be healthy. The estimated blood loss was 750 mL. The patient received 2 U of blood and 1 g of intravenous ceftriaxone intra-operatively. The patient's immediate post-operative condition was satisfactory. All of the surgical specimens were immersed in containers containing appropriate volumes and concentrations of 10% formalin and sent to the histopathology department for histologic diagnoses. After surgery, the patient received intravenous fluid of 1 L of normal saline alternated with 1 L of 5% dextrose water on a 12-hourly schedule, as well as intravenous ceftriaxone (1 g/day) and metronidazole (500 mg every 8 hours for 48 hours). She also received a 30 mg pentazocine intra-muscular injection every six hours for 48 hours. Forty-eight hours after surgery she was placed on oral medications for the next 5 days. She also received hematinics 48 hours after surgery. The urethral catheter was removed 48 hours after surgery. She was continued on oral anti-hypertensive agents after surgery. The stitches were removed on the seventh day after surgery. She was discharged to home on the 10th post-operative day and was scheduled for a two-week follow-up appointment.

**Figure 1:**
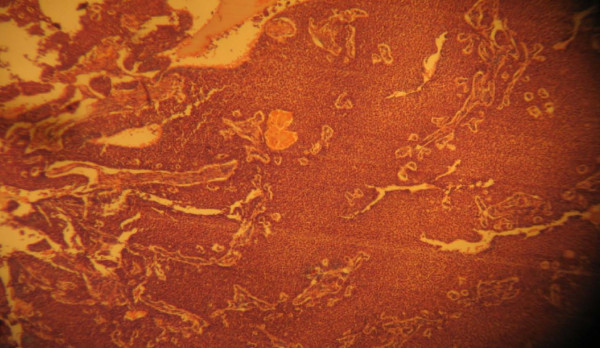
**Photomicrograph showing the ovarian mass**.

**Figure 2:**
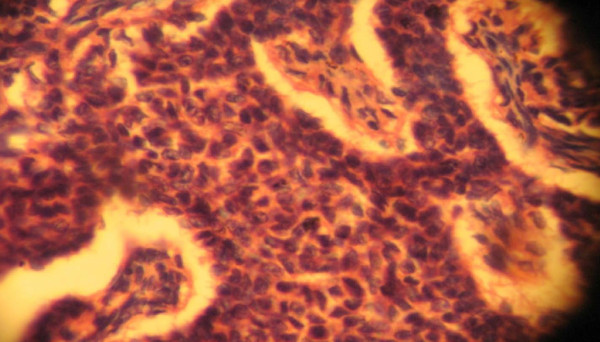
**Photomicrograph showing the ovarian mass**.

**Figure 3:**
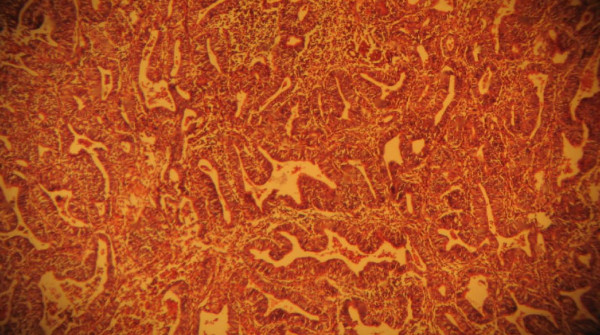
**Photomicrograph showing the uterine mass**.

**Figure 4:**
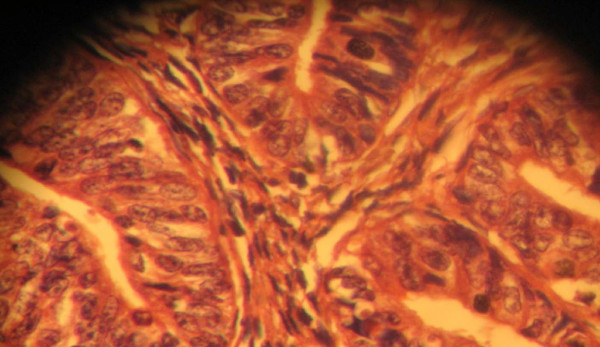
**Photomicrograph showing the uterine mass**.

Macroscopically, the pathology report showed a left ovarian mass. This was a huge, soft to firm, nodular mass weighing 1500 g and measuring 8 cm in its largest dimension. A cut section showed predominantly solid, partly grayish white and partly yellow areas as well as cystic areas filled with blood clots. The uterus was bulky and weighed 700 g. It measured 13.5 cm in its largest dimension. A cut into the endometrial cavity showed several fingerlike friable masses filling the whole uterine cavity. No lesions were observed on the right ovary or the right and left fallopian tubes. Histologic sections of the left ovarian mass showed a granulosa cell tumor containing coffee bean cells growing in different characteristic patterns seen in the tumor. Figures 1 and 2 are photomicrographs of the ovarian mass. Histologic sections of the uterine mass showed a well-differentiated grade 1 endometrioid carcinoma minimally invading the adjacent myometrium. Figures 3 and 4 are photomicrographs of the uterine mass.

At the two-week follow-up visit after surgery, the patient no longer had vaginal bleeding. Her vital signs were normal, and her abdominal wound was well healed. She was last seen six weeks after surgery and had no new complaints. She was advised to continue her follow-up care with the gynecology and cardiology teams at our teaching hospital.

## Discussion

Adult granulosa cell tumors account for approximately 95% of all granulosa cell tumors [1]. We have yet to record any case of a juvenile granulosa cell tumor at our center. Adult granulosa cell tumors occur more often in post-menopausal than pre-menopausal women [1]. This was the case with our patient, who was post-menopausal. If strict criteria for the diagnosis of carcinoma are used and all patients with a granulosa cell tumor, not only those who have undergone endometrial curettage or hysterectomy, are considered, the best estimate of the incidence of associated endometrial carcinomas is under 5% [1]. Our patient presented to our teaching hospital with the first case of granulosa cell tumor and an associated endometrial carcinoma managed in our institution since it was established 20 years ago. We have seen only 21 cases of isolated granulosa cell tumors of the ovary, all of the adult type, since the establishment of the histopathology department at our center. Thus, this puts the incidence of endometrial carcinoma associated with granulosa cell tumor in our center at 4.8%.

Endometrial cancer has been described as consisting of two groups. The first group (type 1, comprising about 80% of the cases) is characterized by well-differentiated tumors that present with localized disease. These patients usually have a favorable outcome [2,3]. The development of type 1 endometrial cancer is associated with excessive estrogen exposure. The risk factors for type 1 endometrial carcinoma include obesity, nulliparity with a history of infertility, late menopause, diabetes mellitus, unopposed estrogen therapy, tamoxifen therapy, and the use of sequential oral contraceptive pills. Excess estrogen from any of these sources produces continuous stimulation of the endometrial lining, which can result in endometrial hyperplasia and can potentially lead to endometrial cancer [4,5].

Ovarian cancers can present as epithelial cell tumors, germ cell tumors, or sex cord-stromal cell tumors [6]. Granulosa cell tumors are classified as germ cell ovarian tumors. These ovarian germ cell tumors may be benign or malignant [6]. Malignant ovarian germ cell tumors are rare, accounting for about 2% to 5% of all ovarian malignancies. However, they are the most common form of ovarian cancer in the first two decades of life. All germ cell tumors arise from the germ cells of the ovary (the oocytes) and are classified according to the type of cell from which they are produced. Ovarian germ cell tumors are classified as dysgerminomas, yolk sac tumors, embryonal carcinomas, polyembryomas, non-gestational choriocarcinomas, teratomas, and mixed germ cell tumors [7]. These cell line origins of germ cell tumors determine the type of proteins they express. Trophoblast-derived tumors produce trophoblastic hormones, particularly human chorionic gonadotropin, but yolk sac tumors produce α-fetoprotein. Both are used in the pre-operative assessment of germ cell tumors and in post-operative follow-up [7].

Teratomas are the commonest type of germ cell tumors that do not produce any particular blood markers. Dysgerminomas are undifferentiated tumors, and, as such, they express the hormonal markers demonstrated by a choriocarcinoma or yolk sac tumor. However, they can also produce other placental hormones, such as placental alkaline phosphatase and lactate dehydrogenase [7].

Granulosa cell tumors cause endometrial cancer by virtue of continuous and unopposed estrogen secretion by the ovary [8]. The microscopic appearance of an endometrioid carcinoma is determined by the grade of the tumor. Grading is based on the architectural pattern of the tumor, its nuclear features, or both. The architectural grade is determined by the extent to which the tumor is composed of solid masses of cells compared with well-defined glands. The nuclear grade is determined by the variation in nuclear size and shape, chromatin distribution, and size of the nucleoli. Grade 1 nuclei are oval and mildly enlarged and have evenly dispersed chromatin. Grade 3 nuclei are markedly enlarged and pleomorphic, with irregular coarse chromatin and prominent eosinophilic nucleoli. The most recent revisions of the International Federation of Obstetricians and Gynecologists (FIGO) Staging System and the World Health Organization Histopathologic Classification of uterine carcinoma recommend that tumors be graded using both architectural and nuclear criteria. The grade of tumors that are architecturally grade 1 or 2 may be increased by one grade in the presence of "notable" nuclear atypia, defined as grade 3 nuclei [9].

Myometrial invasion is an independent predictor of outcome [10,11]. A study of more than 400 patients with clinical stage I endometrioid carcinoma revealed that the 5-year survival rates were 94% when the tumor was confined to the endometrium, 91% when the tumor involved the inner third of the myometrium, 84% when the tumor extended into the middle third of the myometrium, and 59% when the tumor infiltrated the outer third of the myometrium [12]. After surgery, patients are classified as being at low, intermediate, or high risk based on surgical pathologic staging. Patients with grade 1 or 2 tumors that are confined to the endometrium or are minimally invasive are defined as being at low risk and require no further therapy [7,13].

In patients with granulosa cell tumors, estrogen-dependent endometrial cancers can be found, and most of them are well-differentiated endometrioid adenocarcinomas that carry a good prognosis when detected early [11]. This was the case with our patient, who had a well-differentiated, nuclear grade 1 endometrioid adenocarcinoma and FIGO stage 1B disease. This explains why surgery alone was offered to her as a treatment option. Her condition at her follow-up examination six weeks after surgery was satisfactory.

## Conclusion

Because we experienced this case as the first case of an adult granulosa cell tumor associated with a well-differentiated, nuclear grade 1 endometrioid adenocarcinoma in our center, we decided to report it to alert the obstetrics and gynecology community to have heightened clinical suspicion of endometrial carcinoma any time that a diagnosis of granulosa cell tumor is made.

## Consent

Written informed consent was obtained from the patient for publication of this case report and any accompanying images. A copy of the written consent is available for review by the Editor-in-Chief of this journal.

## Competing interests

The authors declare that there is no financial support or relationship that may pose any conflict of interest. The research was funded by the authors.

## Authors' contributions

OCI performed the surgery and assisted in the writing of the manuscript and in the gynecologic work-up of the patient. COU worked on preparing the pathologic slides, assisted in the drafting of the manuscript, and performed PubMed research. GUE performed the gynecologic work-up of the patient and assisted in the writing of the manuscript. ACE performed PubMed research and critically revised the manuscript. All authors read and approved the final manuscript.
